# Nanoemulsion-Based Orodispersible Film Formulation of Guava Leaf Oil for Inhibition of Oral Cancer Cells

**DOI:** 10.3390/pharmaceutics15112631

**Published:** 2023-11-16

**Authors:** Yotsanan Weerapol, Suwisit Manmuan, Tiraniti Chuenbarn, Sontaya Limmatvapirat, Sukannika Tubtimsri

**Affiliations:** 1 Faculty of Pharmaceutical Sciences, Burapha University, Chonburi 20131, Thailand; yotsanan@go.buu.ac.th (Y.W.); suwisit@go.buu.ac.th (S.M.); tiraniti.ch@go.buu.ac.th (T.C.); 2 Department of Industrial Pharmacy, Faculty of Pharmacy, Silpakorn University, Nakhon Pathom 73000, Thailand; limmatvapirat_s@su.ac.th

**Keywords:** orodispersible films, nanoemulsion, guava leaf oil, oral cancer

## Abstract

Among natural sources, guava leaf oil (GLO) has emerged as a potential anticancer agent. However, its limited water solubility poses a significant challenge for its use. Oil-in-water nanoemulsions are used to address the limitation of water solubility of GLO prior to its incorporation into orodipersible films. Nanoemulsions containing GLO:virgin coconut oil (VCO) at a ratio of 50:50 to 70:30 presented a small droplet size of approximately 50 nm and a relatively low zeta potential. GLO:VCO at a ratio of 70:30 was selected for incorporation into sodium alginate film at various concentrations ranging from 1% to 30% *w*/*w*. Tensile strength and elongation at break relied on the concentration of nanoemulsions as well as the internal structure of films. Fourier transform infrared spectroscopy revealed that GLO was compatible with sodium alginate. Film containing 2% *w*/*w* of nanoemulsions (2G_ODF) exhibited effective in vitro antioral cancer activity, with an IC_50_ of 62.49 ± 6.22 mg/mL; furthermore, its anticancer activity showed no significant difference after storage at 25 °C for 1 year. Moreover, 2G_ODF at IC_60_ arrested colony formation and cell invasion. There is also evidence that cell death occurred via apoptosis, as indicated by nuclear fragmentation and positive Annexin-V staining. These findings highlight the potential of orodispersible films containing GLO nanoemulsions as a prospective oral anticancer agent.

## 1. Introduction

Oral cancer, a type of head and neck cancer, is the sixth most prevalent malignancy. Due to its elevated mortality rate and an annual incidence of >300,000 cases worldwide, it has emerged as a significant public health concern [1,2]. Two-thirds of the reported oral cancer cases occur in Asian countries. The prevalence of oral cancer increases with an increase in the age of individuals, with the highest rates of oral cancer observed among individuals >60 years [3]. Other risk factors for oral cancer include tobacco use, alcohol consumption, and betel nut chewing [4]. Notably, in the early stages of oral cancer, patients often do not exhibit any symptoms, leading to neglect of treatment. Furthermore, a delay in diagnosis may elevate the risk of developing advanced stage disease and subsequently increase the mortality rate. Therefore, early treatment and protection, particularly for individuals with high-risk factors, using new natural substances is a favorable choice for mitigating cancer progression [5].

*Psidium guajava* is one of the medicinal trees of the *Myrtaceae* family that is grown in tropical and subtropical regions worldwide. It has been used as a traditional medicine for treating various diseases, including cough, diabetes mellitus, hypertension, microbial infection, inflammation, and cancer [6]. Its essential oil is derived from the fresh or dried leaves of the guava plant via steam distillation or cold-press extraction. Guava leaf oil (GLO) contains various bioactive compounds classified as terpene derivatives, such as β-caryophyllene, limonene, and 1,8-cineole [7,8]. GLO is commonly incorporated as an ingredient in oral care products, thereby aiding in maintaining good oral health. Interestingly, it can arrest the proliferation of various types of cancer cells. Manosoi et al. reported that GLO had the greatest potency against the KB cell line (oral cancer cells), with an IC_50_ value of 0.0379 mg/mL, and it arrested the P388 cell line (murine leukemia cells) with an IC_50_ value of 0.0454 mg/mL [9]. It also exhibited activity against breast and liver cancer cells [10]. In addition, β-caryophyllene, a bioactive compound in GLO, has been reported to exhibit anticancer activity against KB cells by promoting oxidative stress and inducing apoptosis [11]. Nonetheless, the limited compatibility of GLO with water, which corresponds to body fluids, and its inconvenience to use have posed significant constraints on its application as an antioral cancer agent.

Orodispersible films (ODFs) are gaining popularity as a preferred drug delivery system due to their association with excellent patient convenience and compliance. ODFs are ultrathin films designed to rapidly disintegrate or dissolve in the oral cavity upon contact with saliva [12]. They are typically prepared from hydrophilic polymers, such as pullulans, starch, gelatin, pectin, sodium alginate, maltodextrin, hypromellose, and hydroxypropyl cellulose [13,14,15]. ODFs have been used to deliver various active natural substances for the treatment of oral cavity disease, including aqueous extracts of *Plectranthus amboinicus* and *Vaccinium oxycoccos* for inhibiting *Streptococcus mutans* [16] and probiotic bacteria (*Enterococcus faecium*) for eradicating *Candida albicans* [17]. However, no reports on the utilization of ODFs containing essential oils for the prevention and treatment of oral cancer exist. Because of the limited water solubility of GLO, it is challenging to disperse it effectively in an aqueous-based polymer. Hence, the use of a water-compatible carrier to improve the compatibility of GLO with hydrophilic film is imperative.

Nanoemulsions are characterized by their remarkably small droplet sizes, typically ranging from 20 nm to 200 nm [18]. Oil-in-water nanoemulsions have gained popularity as carriers for compounds with limited water solubility, particularly essential oils [19]. Owing to their unique characteristics, nanoemulsions have various advantages in improving physicochemical stability, water compatibility, and biological activities [20]. Therefore, they are considered suitable for inclusion in ODFs.

This study aimed to fabricate an ODF containing a GLO nanoemulsion for the treatment and prevention of oral cancer. Nanoemulsions containing GLO were prepared by incorporating an Ostwald ripening inhibitor, virgin coconut oil (VCO), using the phase inversion temperature technique. Optimized nanoemulsions were incorporated in ODFs, and physicochemical properties, including pH, weight variation, thickness, moisture absorption, disintegration time, mechanical properties, GLO–sodium alginate interaction, and film morphology, were evaluated. In addition, comprehensive assessments to determine the following in oral cancer—cytotoxicity, inhibition of colony formation, anticell invasion effects, and mechanism of cell death—were conducted.

## 2. Materials and Methods

### 2.1. Materials

The following ingredients were used in this study: GLO (Lot No. 20230222, Chemipan Coporation, Bangkok, Thailand), β-caryophyllene standard (Lot No. 102429598, Sigma-Aldrich, St. Louis, MO, USA), VCO (Lot No. SS54/06177-01, Tropicana Oil, Nakhon Pathom, Thailand), sodium alginate (Lot No. H052204110-01, T.C. Sathaporn, Bangkok, Thailand), glycerin (Lot. No. 1411181868, Ajax Finechem Pty Ltd., Auckland, New Zealand), and Cremophor RH40 (PCO40; Lot No. 3069747G0, PC Drug Center, Bangkok, Thailand). Human oral squamous cancer cells (KON; Lot No. 01262007) were purchased from the Japanese Collection of Research Bioresources Cell Bank, Tokyo, Japan. The 3-(4,5-dimethylthiazol-2-yl)-2,5 diphenyl tetrazolium bromide (MTT; Lot No. 39H5076) and dimethyl sulfoxide (DMSO; Lot No. #SHBH5546v) were purchased from Sigma-Aldrich, St. Louis, MO, USA. Fetal bovine serum (FBS; Lot No. RH20220005) was purchased from Cytiva, Marlborough, Massachusetts, USA. Phosphate buffer saline (PBS; Lot No. 29420002) and Dulbecco’s modified eagle medium (DMEM; Lot No. 23621012) were obtained from Corning, New York, NY, USA. All other chemicals and reagents used in this study were of analytical grade.

### 2.2. Preparation of Nanoemulsions

As reported in previous studies, essential oils without fixed oil incorporation were unable to generate nanoemulsions with a droplet size in the nano range. Fixed oil was necessary to prevent Ostwald ripening during emulsion formation [21,22]. To achieve the smallest droplet size, the ratio of GLO to VCO in the range of 50:50 to 100:0 was analyzed before ODF fabrication. Nanoemulsions containing GLO were prepared using the phase inversion temperature method. VCO and PCO40 were heated to 62 °C. As the temperature approached 62 °C, GLO was combined with the heated VCO and PCO40 to create the oil phase. The water phase was subjected to a temperature of 65 °C. Then, the water and oil phases were blended using a homogenizer (T25D, IKA, Staufen, Germany) for 5 min at a speed of 3800 rpm. The obtained nanoemulsions were comparatively evaluated in terms of droplet size and zeta potential before and after undergoing a temperature-cycling for six cycles (with each cycle consisting of 45 °C for 24 h followed by 4 °C for 24 h).

### 2.3. Droplet Size Determination

Using dynamic light scattering (Zetasizer Nano-ZS, Malvern Instruments, Worcestershire, UK) with the detector position at either 175° or 90°, droplet sizes of 1 mL of GLO nanoemulsion were measured with a disposable capillary cell at 25 °C. The average and standard deviations were calculated and recorded (n = 3).

### 2.4. Zeta Potential Assesment

Zeta potential was assessed using a zetasizer (Zetasizer Nano-ZS, Malvern Instruments, Worcestershire, UK). Approximately 1 mL of nanoemulsion was introduced into a disposable capillary cell. The samples were then run in triplicate at 25 °C, and the means with SD were noted.

### 2.5. Fabrication of ODF Containing GLO Nanoemulsions

After obtaining nanoemulsions with the smallest droplet size and good physical stability (indicated by no change in droplet size after temperature cycling test), a film containing GLO nanoemulsions was fabricated using the solvent casting method. Different concentrations of nanoemulsions (1%, 2%, 5%, 10%, 20%, and 30% *w*/*w*) were mixed with 1% *w*/*w* of sodium alginate powder, and the weight was adjusted with purified water until 100% was reached. Film solutions were individually poured onto the plate and left to dry at room temperature for 24 h. The obtained ODFs containing several nanoemulsion concentrations—1% *w*/*w* (1G_ODF), 2% *w*/*w* (2G_ODF), 5% *w*/*w* (5G_ODF), 10% *w*/*w* (10G_ODF), 20% *w*/*w* (20G_ODF), and 30% *w*/*w* (30G_ODF)—were comparatively investigated.

### 2.6. pH Measurements

The pH of ODF solutions was measured to assess their suitability for the oral cavity using a pH meter (SevenMulti, Mettler Toledo, Columbus, OH, USA). Three samples were measured and presented as mean ± SD.

### 2.7. Evaluation of Weight Variation

ODFs with a 4 cm^2^ surface area from several experiments were chosen and weighed on an analytical balance (QUINTIX224-1S, Sartorius, Göttingen, Germany). The average weight of five films was calculated and recorded as mean ± SD.

### 2.8. Evaluation of Thickness Uniformity

The thickness of the ODFs was assessed using dial thickness gauges (Oxford precision, Leicester, UK). The values were recorded as mean ± SD (n = 3).

### 2.9. Moisture Absorption Assessment

ODFs from three experiments were introduced into a desiccator with a saturated potassium chloride solution at 80% relative humidity and left for 24 h at room temperature. Afterward, each film was individually weighed, and the percentage of moisture absorption was computed using the below equation and recorded as mean ± SD.

$$\mathrm{Moisture}\;\mathrm{absorption}\;\%=100\;\times \;\frac{\mathrm{Final}\;\mathrm{weight}\;-\;\mathrm{Initial}\;\mathrm{weight}}{\mathrm{Initial}\;\mathrm{weight}}$$

### 2.10. Determination of Disintegration Time

The disintegration time of ODFs was determined using the modified method of Arya et al. [23]. A 1 cm^2^ section of the ODFs was immersed in 10 mL of phosphate buffer with pH 6.75 at 37 °C. The disintegration time was when the film began to break apart. The tests were performed in triplicate, and values are presented as mean ± SD.

### 2.11. Analysis of Mechanical Properties

A texture analyzer (TA.XT plus, Stable Micro Systems, Surrey, UK) was used to measure the tensile strength and elongation at break of the ODFs containing different concentrations of GLO nanoemulsions. Three ODFs from each concentration were cut into 1 × 10 cm^2^ pieces. To achieve a gap length of 1 cm, the substrate was then glued from top to bottom. The ODF was then pulled with a test speed of 1 mm/s. The maximum force and distance before film breakage were noted and calculated as tensile stretch and elongation at break using the following equations:

$$\mathrm{Tensile}\;\mathrm{stress}\;(N/{\mathrm{mm}}^{2})=\frac{Force\;at\;breakage\;(N)}{Film\;thickness\;(mm)\times Film\;width\;(mm)}$$

$$\mathrm{Elongation}\;\mathrm{at}\;\mathrm{break}\;(\%)=100\;\times \;\frac{Increase\;in\;the\;ODF\;length}{Initial\;length\;of\;the\;ODF}$$

### 2.12. Characterization of Infrared Spectroscopy

To determine the interaction among molecules within the ODFs, the infrared spectra of the films in attenuated total reflectance (ATR) mode were measured. The samples were analyzed using an ATR crystal and scanned in the wave number range of 4000–400 cm^−1^, with a resolution of 2 cm^−1^ on diamond crystal ATR Fourier transform infrared (FTIR) spectroscopy (Niclolet 6700, Thermo Electron Corporation, Waltham, MA, USA).

### 2.13. Scanning Electron Microscopy (SEM)

The microstructure of ODFs was analyzed via SEM with back-scattered electrons (Mira3, Tescan, Brno, Czech Republic). An 8 × 8 mm^2^ of ODFs was fixed on a double adhesive carbon tape and placed on an aluminum tub. The samples were coated with gold under vacuum before examination. The images were scanned at an acceleration voltage of 10–15 kV. Excessive oils in 20G_ODF and 30G_ODF were removed with lint free paper (Kimwipes ™) before investigation.

### 2.14. Quatification of β-Caryophyllene

The β-caryophyllene was labeled as a marker for this study because it has been documented against cancer cells [24]. The concentration of β-caryophyllene in GLO and ODFs was analyzed using gas chromatography (GC; 7890A, Agilent Technologies, Santa Clara, CA, USA) equipped with a flame ionization detector and an HP-5 capillary column (30 m × 0.25 mm, 0.32-μm film thickness, and temperature programmed as follows: 100 °C–140 °C at 10 °C/min, 140 °C–180 °C at 2.5 °C/min, and 180 °C–200 °C at 20 °C/min). Helium, with a flow rate of 1.0 mL/min, was used as the carrier gas. The front inlet and detector temperatures were set at 240 °C and 280 °C, respectively. The sample was injected using a splitting method, with a split ratio of 80:1 [25]. Before analysis, the GLO was dissolved in absolute ethanol. The ODF was dissolved in water and freeze dried (Scanvac, Labogene, Lynge, Denmark) prior to dissolving in absolute ethanol. The percentage of β-caryophyllene in GLO and a representative ODF was calculated from the peak area on its standard curve, which was fabricated in the range of 5–50 µg/mL with R^2^ of 0.9992 (Supplementary Figure S1). The β-caryophyllene content was determined using the following equation:

$$\beta \text{-}\mathrm{caryophyllene}\;\mathrm{content}\;(\text{µ}g/g)=\frac{\beta \text{-}caryophyllene\;content\;in\;sample}{Weight\;of\;sample}$$

### 2.15. Cytotoxicity Test

The cytotoxicity of β-caryophyllene, GLO, and optimized ODF was determined using MTT assay. Before the experiment, KON cells or MRC-5 cells (normal fibroblast cells) were cultured in DMEM supplemented with 10% FBS and 0.01% glutamine. The cells were then seeded into a 96-well plate at a density ranging from 5 × 10^3^ to 1 × 10^4^ cells/well, following a 24 h culture at 37 °C in a 5% CO_2_ incubator (KBF-240, Binder, Tuttlingen, Germany) before introducing with samples. After the 24 h incubation, the culture medium was aspirated, and each well was treated with 50 µL of MTT solution (0.5 mg/mL), which was then incubated for 3 h in the dark. A microplate reader (Fluostar Omega, BMG Labtech, Ortenberg, Germany) was used to assess the absorbance of each well at 570 nm after adding 50 µL of DMSO. To ascertain the IC_50_ value, i.e., the film concentration that can arrest 50% of cancer cells, a correlation was established between the percentage of cell viability and the concentration of ODFs. Cell viability and cytotoxicity were determined using the following equation:

$$\mathrm{Cell}\;\mathrm{viability}\;(\%)=100\;\times \;\frac{Mean\;absorbance\;of\;treated\;cell-Mean\;absorbance\;of\;blank}{Mean\;absorbance\;of\;untreated\;cell-Mean\;absorbance\;of\;blank}$$

### 2.16. Stability Test

Three samples of ODFs were maintained in a tight container and protected from light at 25 °C for 1 year. Subsequently, these samples were assessed for their tensile strength, β-caryophyllene content, and anticancer activity. The recorded values are presented as mean ± SD (n = 3).

### 2.17. Colony Formation Assay

In each well of a six-well plate, approximately 1000 KON single cells were cultivated in DMEM with 10% FBS and 0.01% of glutamine at 37 °C and 5% CO_2_ for 24 h. The optimized formulation of ODF was placed into the well for 2, 5, 10, 15, and 30 min. The medium containing ODF was subsequently removed, and each well was rinsed twice with PBS. Fresh medium was added into each well and incubated at 37 °C and 5% CO_2_ for 10–14 days. Colonies were stained with 0.5% crystal violet in PBS for 30 min at ambient temperature after fixing them with methanol. Water was used to repeatedly wash away the excessive crystal violet stain. The colonies of the ODF treatment group were counted, and the percentage of proportional colony formation was calculated by the following equation:

$$\mathrm{Proportional}\;\mathrm{of}\;\mathrm{colony}\;\mathrm{formation}\;(\%)=100\;\times \;\frac{Number\;of\;colonies\;after\;treated\;with\;sample}{Number\;of\;colonies\;of\;negative\;control}$$

### 2.18. Cell Metastasis Assay

The antimetastasis property of the ODFs was assessed using a cell invasion assay. A 24-well plate was filled with 40 µL diluted Matrigel, which was then left to polymerize for 24 h. Then, 1 × 10^3^ of KON cell suspension and DMEM (negative control) or optimized ODF at IC_60_ or PCO40 solution were added to the superior part of the well with a pore size of 8 µm. PCO40 was selected to compare the antioral cancer activity with ODF because it can induce cell death by cell membrane lysis [26]. In the lower chamber, DMEM (500 µL) with 10% FBS was added. After 48 h, the medium was extracted from both parts, and invading cells in the Matrigel layer were fixed in ice-cold methanol (1 mL) at ambient temperature for 15 min. Simultaneously, noninvading cells were eliminated from the superior part using a cotton swab. After staining the invading cells with crystal violet (500 µL) for 30 min, they were washed with PBS to eliminate excess crystal violet until the chamber bottom was clear. Subsequently, the number of KON cell colonies was counted from three experiments, and the invasiveness was recorded as a percentage of proportional invasiveness using the following equation:

$$\mathrm{Proportional}\;\mathrm{invasiveness}\;(\%)=100\;\times \;\frac{Number\;of\;cells\;invaded\;in\;treated\;cells}{Number\;of\;cells\;invaded\;in\;negative\;control}$$

### 2.19. Apoptosis Assay

#### 2.19.1. Analysis of Nuclear Fragmentation

Approximately 1 × 10^4^ cells/well were cultivated on sterile coverslips in a six-well plate at 5% CO_2_ and 37 °C in a CO_2_ incubator (KBF-240, Binder, Tuttlingen, Germany) for 24 h. ODF at IC_60_ or PCO40 solution was applied to the KON cells, and DMEM was designated as the negative control. After 24 h of incubation, the KON cells were gently washed using a PBS solution and fixed with paraformaldehyde (4% *v*/*v*) for 15 min. Triton-X 100 (0.2% *v*/*v*) was used to permeabilize the fixed cells before staining them with 5 µL of Hoechst 33258 in the dark condition. After 10 min, excessive Hoechst 33258 was washed off using PBS. Apoptotic cells were determined using an inverted fluorescence microscope (ELIPSR Ts2, Nikon, Tokyo, Japan).

#### 2.19.2. Flow Cytometry Assay

Approximately 1 × 10^6^ cells/well of KON cells were added into a six-well plate for 24 h. The optimized formulation of ODF at the concentration of IC_60_ or PCO40 solution or DMEM (negative control) was included into each well. After 16–20 h, the cells were collected and stained with an Annexin-V-FITC apoptosis detection kit I (BD Biosciences, San Diego, CA, USA). The stained cells from the three experiments were examined using a flow cytometer (CytoFlex, Beckman Coulter, Brea, CA, USA) after incubation for 15 min at ambient temperature in the dark. Live cells were designated as Annexin−/PI−, early apoptotic cells as Annexin+/PI−, late apoptotic cells as Annexin+/PI+, and necrotic cells as Annexin−/PI+.

### 2.20. Statistical Analysis

SPSS 10.0 for Windows (SPSS Inc., Chicago, IL, USA) was used for statistical analysis. One-way analysis of variance followed by Tukey’s test and paired *t*-test was used to evaluate the results. *p*-values less than 0.05 were used to indicate statistical significance.

## 3. Results and Discussion

### 3.1. Droplet Size and Zeta Potential of GLO Nanoemulsions

**Figure 1:**
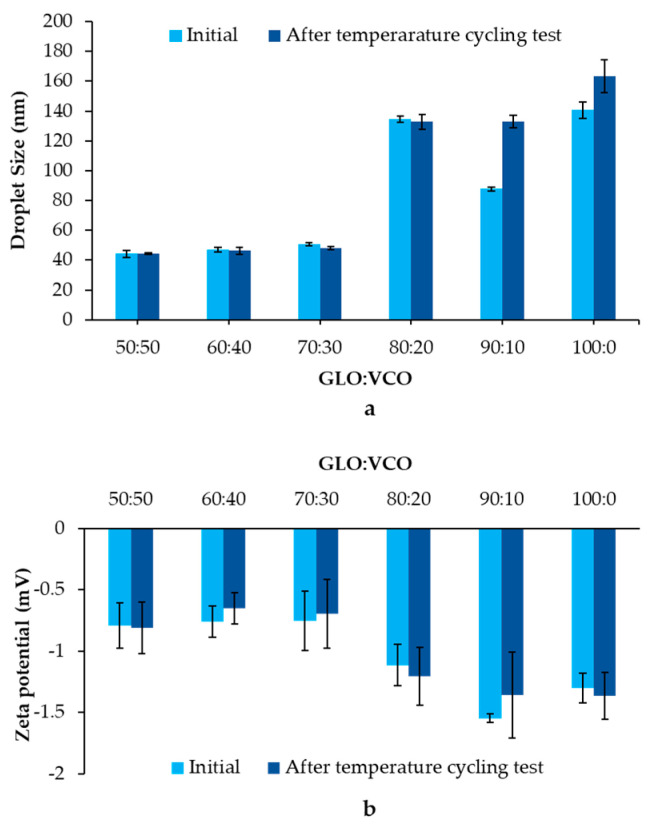
Droplet size (**a**) and zeta potential (**b**) of nanoemulsions produced using various ratios of GLO:VCO before and after the temperature-cycling test. The results are depicted as mean ± SD (n = 3).

Fixed oil is a crucial component for producing essential oil nanoemulsions via retardation of Ostwald ripening, as it possesses nanosized droplets and excellent stability [27]. To fabricate stable nanoemulsions, VCO, a fixed oil, was chosen as the key component. The droplet size and zeta potential of nanoemulsions produced by several ratios of GLO:VCO were evaluated before and after the temperature-cycling test, as depicted in Figure 1a and Figure 1b, respectively. Nanoemulsions prepared from GLO:VCO of 50:50 to 70:30 presented a small droplet size of approximately 50 nm, whereas the other GLO:VCO ratios resulted in a larger droplet size (80–150 nm). These results were consistent with those of previous studies reporting that the droplet size of essential oil nanoemulsion depends on the oil phase composition, especially the ratio of the essential oil to fixed oil [21,22]. After the temperature-cycling test, the droplet size of the GLO:VCO, at ratios ranging from 50:50 to 80:20, was not significantly different from the initial measurements, whereas 90:10–100:0 tended to increase after storage. This result suggested high stability of the 50:50 to 80:20 GLO:VCO.

Regarding the zeta potential, nanoemulsions ranging from 50:50 to 70:30 GLO:VCO presented a relatively low value of approximately −0.7 mV. This might be due to the complete covering of nonionic surfactant (PCO40) around oil droplets. The result was consistent with that of our previous report disclosing that the stable nanoemulsion presented a low zeta potential close to zero when using a nonionic surfactant as an emulsifier [22]. In addition, the zeta potential tended to increase with the increasing of VCO concentration. The presence of a negative charge from the carboxylic group in VCO might contribute to these phenomena. The zeta potential depended on VCO concentration, resulting in a high relative zeta potential value in the 50:50 and a low relative zeta potential in the 70:30. Notably, the relative zeta potential of 80:20–90:10 was higher than that of 50:50–70:30. This might be due to a decrease in the neutral charge of GLO within the droplet. The lower VCO concentration led to a decrease in GLO entrapment efficacy, thereby enhancing water solubility of GLO. Consequently, the negative charge of the VCO became more prominent. Likewise, 90:10 showed a higher zeta potential value, although it comprised a lower VCO fraction than 80:20. At 100:0, the zeta potential value relatively decreased due to the increase in the neutral charge of GLO without the VCO negative charge [28,29]. Furthermore, the zeta potential of all formulations differed insignificantly from the initial value after the temperature-cycling test.

These results suggest that the presence of an optimal concentration of the fixed oil, which acts as an Ostwald ripening inhibitor, led to the production of small droplet sizes and enhanced the stability of the nanoemulsions by counteracting Ostwald ripening. Thus, due to the small size of nanoemulsions, low zeta potential, good stability, and high GLO loading, GLO:VCO at 70:30 is preferred for inclusion in ODFs.

### 3.2. pH

Several concentrations of nanoemulsions containing GLO:VCO at 70:30 ranging from 1% to 30% were incorporated into 1% *w*/*w* of sodium alginate. Before water evaporation from formulations, the pH of the ODF solutions was examined using a pH meter. As depicted in Table 1, the pH of the ODF solutions ranged from 5.46 to 6.24. A reduction in pH was found when the concentration of the nanoemulsions increased due to the acidic property of GLO nanoemulsions (pH = 3.67 ± 0.01). The pH of all ODF solutions was lower than that of the oral cavity, which ranged from 6.8 to 7.8 [30]. However, the pH of all ODF solutions was higher than that of enamel dissolution (pH 5.5) [31]. This result suggested that all ODFs can be used in the oral cavity without damaging the dental enamel.

### 3.3. Average Weight and Film Thickness

After obtaining ODFs, the ODFs were cut into 2 × 2 cm^2^ prior to evaluating their weight and film thickness. As depicted in Table 1, the weight of the ODFs ranged from 18.3 ± 2.8 µg to 46.5 ± 7.8 µg. The thickness of the ODFs ranged from 41.3 ± 9.0 µm to 121.0 ± 9.0 µm, which was in the range of typical ODF thickness (25–100 µm), except for 30G_ODF [32]. Notably, the weight and thickness of the ODF tended to increase with an increase in the nanoemulsion concentration. This phenomenon could be attributed to the higher level of entrapped GLO, VCO, and PCO40 in the ODFs when the nanoemulsion concentration was increased [33].

### 3.4. Moisture Absorption

Moisture absorption significantly influences film characteristics, including friability, mechanical strength, disintegration, and dissolution [34]. Films with low moisture absorption exhibit good stability and durability in high-humidity environments. As illustrated in Table 1, the moisture absorption of the ODFs ranged from 10.65% ± 1.20% to 49.29% ± 6.18%. High moisture absorption was attributed to the nature of sodium alginate, which is reportedly a superabsorbent material [35]. These findings suggested that ODFs should be kept in tight containers. Furthermore, moisture absorption decreased when the nanoemulsion concentration increased. These findings correlated with those of Fasihi et al., who reported that increasing essential oil nanoemulsions might raise the tortuosity path of the polymer chains, decreasing water diffusion [36].

### 3.5. Disintegration Time

Table 1 presents the disintegration times of ODFs. The disintegration time of each formulation ranged from 12.75 ± 2.60 s to 22.75 ± 4.50 s. All formulations had a disintegration time of less than 60 s, which was the ideal disintegration time of ODFs [37]. Furthermore, the disintegration time increased with an increase in the nanoemulsion concentration. These results correlated with those of moisture absorption. The prolonged disintegration time might be due to an increase in the tortuosity path of the polymer chains.

### 3.6. Mechanical Strength of the OFDs

**Figure 2:**
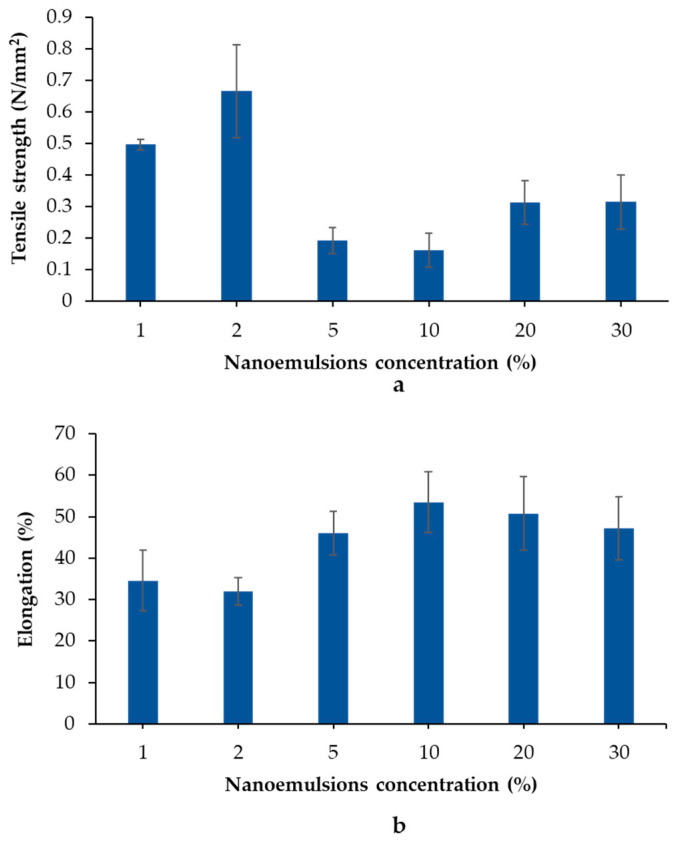
Tensile strength (**a**) and elongation (**b**) of ODFs. The results are depicted as mean ± SD (n = 3).

Tensile strength and elongation are general parameters used to define the mechanical property of ODFs. The terms tensile strength and elongation at break refer to the resistance to tension forces and the capacity of a film to stretch, respectively. Figure 2 shows the mechanical characteristics of the ODFs containing nanoemulsions ranging from 1% *w*/*w* to 30% *w*/*w*. The tensile strength of the sodium alginate film was 0.11 ± 0.01 N/mm^2^. After incorporation of GLO nanoemulsions, the tensile strength increased up to that of 2G_ODF and then decreased at higher loadings. The tensile strength began to increase again when the GLO nanoemulsions were increased to >20% *w*/*w* (Figure 2a). According to previous reports, hydrophobic compounds can increase the interaction force between the polymer chains [38]. This could explain the observed increase in tensile strength values when incorporating GLO nanoemulsions at concentrations <2%. However, high oil incorporation probably disrupted the cohesive force of the polymer, leading to a reduction in tensile strength, as observed in 5G_ODF and 10G_ODF [39]. When GLO nanoemulsions >20% *w*/*w* were incorporated, the oil droplets underwent coalescence and segregation from the polymer during the film-drying process, thereby reducing the disruptive cohesive forces of the polymer [40].

Furthermore, GLO nanoemulsions affected the percentage of elongation of the ODFs (Figure 2b). Contrary to tensile strength, the elongation percentage decreased from 34.59% ± 7.24% to 31.92% ± 3.33% after incorporation of 1–2% *w*/*w* GLO nanoemulsion. With increasing GLO nanoemulsion (5–10%), the percentage increased from 46% to 53% and decreased after incorporating GLO nanoemulsion >20% *w*/*w*. As discussed in tensile strength, the increased interaction force between polymer molecules after loading GLO nanoemulsion <2% *w*/*w* reduced the plasticizing effect of the GLO nanoemulsion, leading to a low elongation percentage. Subsequently, when an appropriate concentration of GLO nanoemulsion is introduced, the connections between the polymer networks may be reduced. This allowed an increase in the plasticizing effect and elongation percentage; this property was directly correlated with the concentration of GLO nanoemulsion, as observed in 5–10% *w*/*w* [41]. After loading >20% *w*/*w* of GLO nanoemulsion, the aggregation of oil droplets rather than uniform insertion in sodium alginate film might cause a reduction in the elongation percentage [42].

### 3.7. FTIR

**Figure 3:**
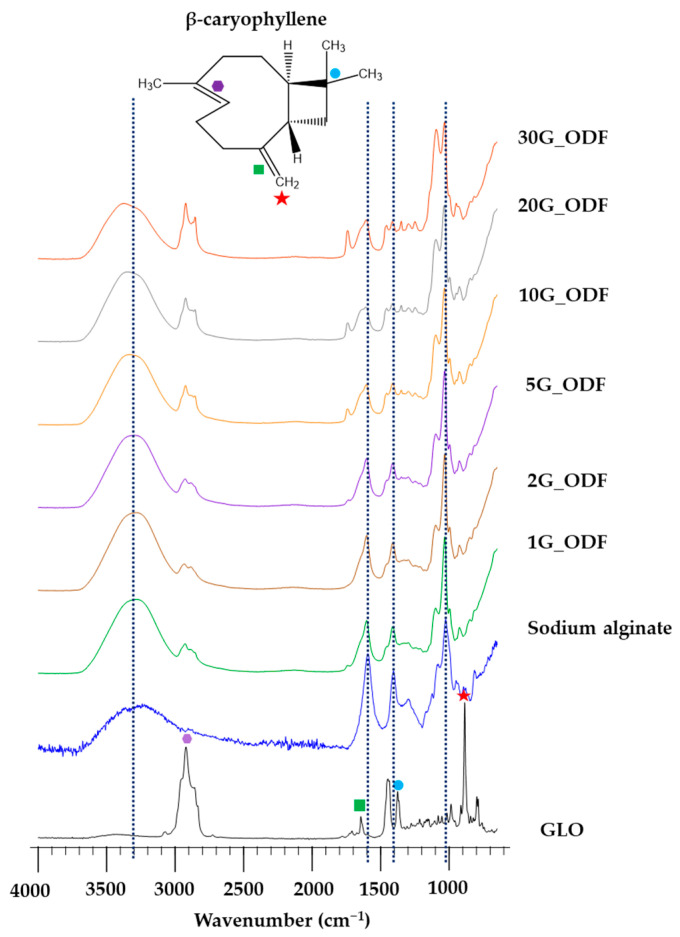
IR spectrum of GLO, sodium alginate, and ODFs.

ATR-FTIR spectroscopy was used to analyze the compatibility of GLO and sodium alginate. The FTIR spectrum in Figure 3 corresponds to GLO, sodium alginate, and ODFs. GLO, with β-caryophyllene as a reference compound, possessed the following peaks: C–H stretching at 2910 cm^−1^ (hexagonal purple), C=C stringing at 1654 cm^−1^ (green square), C–H deformation at 1378 cm^−1^ (blue circle), and C–H deformation at 891 cm^−1^ (red star). Sodium alginate presented a peak from 3634 to 2442 cm^−1^ and at 1590 cm^−1^, 1400 cm^−1^, and 1027 cm^−1^, which are presented as black lines. After incorporating GLO nanoemulsions, the peak corresponding to C–H stretching at 2910 cm^−1^ was observed in all ODFs, whereas the peaks corresponding to C=C stringing at 1654 cm^−1^, C–H deformation at 1378 cm^−1^, and C–H deformation at 891 cm^−1^ disappeared. This might be due to the low concentration of GLO. However, the remarkable peak of sodium alginate did not exhibit any shifting. These results suggested that there was no interaction between GLO and sodium alginate. It can be concluded that GLO was compatible with sodium alginate.

### 3.8. SEM

**Figure 4:**
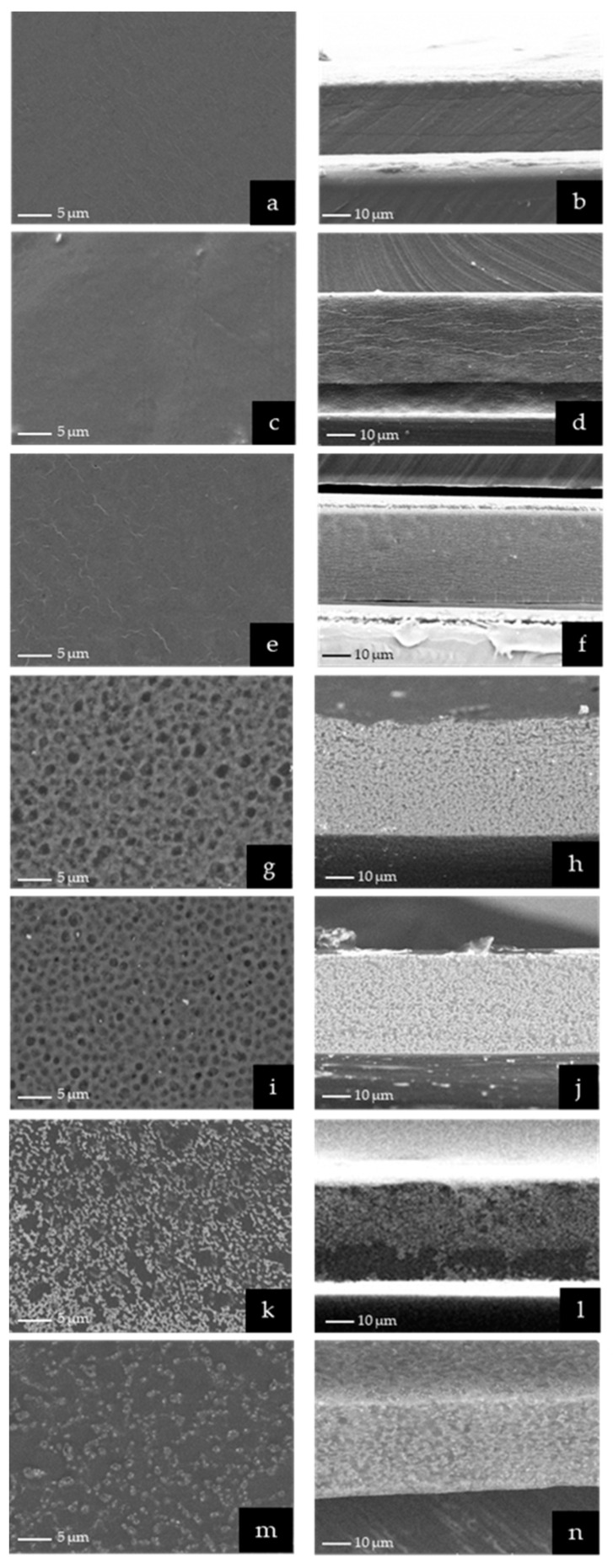
Surface SEM images of sodium alginate film (**a**), 1G_ODF (**c**), 2G_ODF (**e**), 5G_ODF (**g**), 10G_ODF (**i**), 20G_ODF (**k**), and 30G_ODF (**m**) and cross-section SEM images of sodium alginate film (**b**), 1G_ODF (**d**), 2G_ODF (**f**), 5G_ODF (**h**), 10G_ODF (**j**), 20G_ODF (**l**), and 30G_ODF (**n**).

To understand the arrangement of oil droplets inside the film and verify the aggregation of GLO nanoemulsions during the drying process, the microstructure of ODFs was examined via SEM. As presented in Figure 4, the SEM images (surface and cross-section) showed diverse microstructures at different levels of GLO nanoemulsions. The ODFs containing nanoemulsions had coarser microstructures than the sodium alginate films. The uniform and absence of oil aggregation was observed in 1G_ODF and 2G_ODF, indicating that GLO nanoemulsions droplets were evenly distributed and incorporated within the sodium alginate network. The presence of a uniformly porous microstructure was notably observed in 5G_ODF and 10G_ODF. An increase in the concentration of GLO nanoemulsions, i.e., more than 20% (20_ODF and 30G_ODF), resulted in observable aggregation of oil droplets. Oil droplets had a tendency to coalesce rather than embed in sodium alginate films. The reduction of oil droplets insertion led to certain regions of the films exhibiting greater dense structure than that of 5G_ODF and 10G_ODF. These results correlated with a previous report that revealed the effect of nanoemulsion concentrations on the microstructure of films [33]. The different microstructures of ODFs loaded with several concentrations of nanoemulsion also clarified the variations of mechanical properties. High tensile strength observed in 1G_ODF and 2G_ODF was a result of compact microstructure. The presence of a hollow structure resulted in a decrease of tensile strength, as observed in 5G_ODF and 10G_ODF [43]. In comparison to 5G_ODF and 10G_ODF, 20G_ODF and 30G_ODF exhibited larger tensile strength due to the migration of oil droplets to the film surface.

### 3.9. β-Caryophyllene Content

The 2G_ODF, 5G_ODF, and 10G_ODF were selected as representative ODFs for this experiment due to high GLO loading without oil separation. As illustrated in Table 2, the β-caryophyllene content in GLO was found to be 176,950.98 ± 239.55 µg/g or 17.69% ± 0.02% *w*/*w*, correlating with Sherweit et al. [28]. The β-caryophyllene contents of 2G_ODF, 5G_ODF, and 10G_ODF were 129.51 ± 3.58 µg/g, 220.82 ± 12.04 µg/g, and 309.58 ± 15.92 µg/g, respectively. The limited size and thickness of the film base could be responsible for the low drug content [44]. However, due to the low drug content, it is necessary to conduct further investigation of anticancer activity to determine its effectiveness.

### 3.10. MTT Assay

The anticancer activity of GLO and ODFs against oral cancer was determined using an MTT assay. 2G_ODF, 5G_ODF, and 10G_ODF were selected as representative films in this experiment. Table 3 shows that the IC_50_ values of β-caryophyllene, GLO, 2G_ODF, 5G_ODF, and 10G_ODF were 3.73 ± 0.58 µg/mL, 11.65 ± 2.61 µg/mL, 62.49 ± 6.22 mg/mL, 24.11 ± 1.22 mg/mL, and 15. 29± 2.04 mg/mL, respectively. The PCO40 solution (a concentration equal to that used in 2G_ODF at IC_50_) exhibited a lack of antioral cancer activity (percentage of cell viability = 109.48% ± 11.78%), indicating that the anticancer activity is a direct consequence of GLO. In addition, the anticancer activity of ODF depended on GLO concentration, which 10G_ODF exhibited the most potently against oral cancer cells. When considering the antioral cancer activity of β-caryophyllene and GLO, it was observed that the anticancer activity of GLO was three times lower than that of β-caryophyllene, although β-caryophyllene constituted only one-fifth or 17.69% ± 0.02% of GLO. This suggested that the antioral cancer activity resulted from the synergistic effects of several active substances in GLO. According to the US National Cancer Institute (NCI), GLO was classified as a cytotoxicity agent due to its IC_50_ value of <20 µg/mL [45]. However, the limited size and thickness of the film base could be responsible for the drug loading, decreasing the anticancer activity after incorporating GLO into ODF [44].

In consideration of its toxicity to normal cells, the IC_50_ of 2G_ODF against MRC-5 cells was found to be 97.20 ± 6.31 mg/mL. This indicates that the concentration of 2G_ODF used to inhibit oral cancer cells did not exhibit toxicity towards normal cells. A similar re-sult was reported by Braga et al., who found that the ethanolic extract of the *P. guajava* leaf was not toxic to normal cells [46]. Therefore, it can be concluded that the immediate release of 2G_ODF in the mouth does not result in any toxic effects on oral tissue.

### 3.11. Assessment of ODF Stability

2G_ODF was selected as a representative ODF for stability test because it exhibited good physical properties with maximum loading. As presented in Table 4, after storage at 25 °C for 1 year, the tensile strength of 2G_ODF was not significantly different compared with the initial measurements, suggesting that 2G_ODF exhibited good physical stability. Furthermore, 2G_ODF displayed high chemical stability, as indicated by the lack of significant change in β-caryophyllene content after storage at 25 °C for 1 year. With regard to biological stability, after storage at 25 °C for 1 year, the ODF still presented anticancer activity with an IC_50_ of 65.53 ± 5.12 mg/mL, which was not significantly different from that during the initial days. The results suggested that ODF possessed good biological stability. These might be results from excipients in the ODF. VCO and sodium alginate film can help protect GLO against environmental change, leading to the increased stability of bioactive components and the maintenance of biological activities [47].

### 3.12. Colony Formation Assay

**Figure 5:**
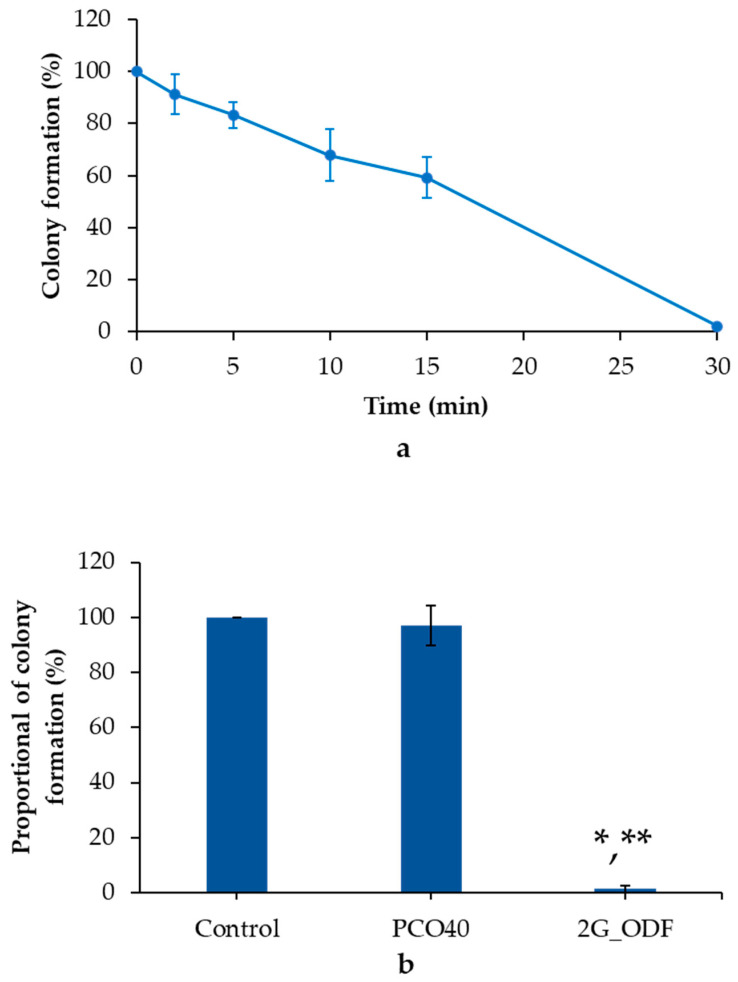
Colony formation after treatment with IC_60_ of 2G_ODF for 2, 5, 10, 15, and 30 min (**a**) and proportion of colony formation after treatment with IC_60_ of 2G_ODF and PCO40 solution (equivalent to that used for 2G_ODF) for 30 min (**b**). * denotes a significant difference (*p* < 0.05) compared with the control. ** denotes a significant difference (*p* < 0.05) compared with the PCO40 solution. The results are presented as mean ± SD (n = 3).

The colony formation assay is one of the in vitro cell viability assays that considers the individual cell capacity to develop into a colony. In this study, the KON colony was treated with 2G_ODF for 2, 5, 10, 15, and 30 min to assess its effectiveness at different time intervals. As illustrated in Figure 5a, 2G_ODF exhibited a time-dependent pattern. The viability of the KON colony was reduced to 80% after exposure to 2D_ODF for 5 min. The gradual decrease to approximately 50% was observed for 15 min. The strongest inhibition was detected after treatment for 30 min.

When exposed to saliva, it was assumed that 2G_ODF transformed to small droplets and subsequently penetrated the pass-through cancer cell membrane. After accumulation of GLO nanoemulsion within the oral carcinoma for 5 min, it induced cell death. However, the concentration of GLO within the oral carcinomas was too low; therefore, it was incompletely inhibited. As time increased, the increase in accumulated GLO enhanced antioral cancer activity, and complete inhibition occurred after 30 min.

The anticolony formation of 2G_ODF at 30 min was also comparatively evaluated with the PCO40 solution and negative control. The results showed significant differences among 2G_ODF, PCO40 solution, and the negative control treatment group at a 95% confidence interval. In the 2G_ODF treatment group, colony formation was rarely detected, with a proportional colony formation of 1.33% ± 1.15% compared with negative control. On the contrary, a rapid increase in the development of larger colonies in the PCO40 solution treatment group was observed, with an in-proportion of colony formation of 96.99% ± 7.28%, as shown in Figure 5b. These findings suggest that 2G_ODF not only inhibited cell proliferation but also effectively arrested colony formation in a time-dependent manner. This characteristic was a direct result of GLO, regardless of surfactant.

### 3.13. Determination of Antimetastasis Activity

**Figure 6:**
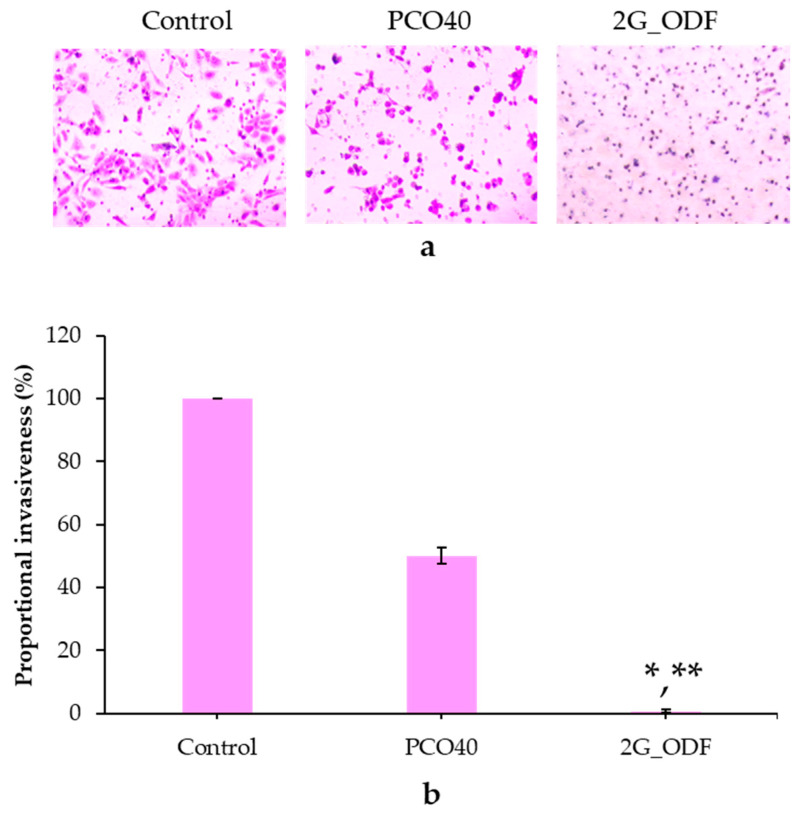
Antimetastatic assay: invading cells (**a**) and proportional invasiveness (**b**) after treatment with IC_60_ of 2G_ODF and PCO40 solution (equivalent to that used for 2G_ODF). * denotes a significant difference (*p* < 0.05) compared with the control. ** denotes significant difference (*p* < 0.05) compared with the PCO40 solution. The results are presented as mean ± SD (n = 3).

Metastasis involves cancer cells spreading from the original part to other areas of the body, most generally through the lymph system or bloodstream, resulting in the progressive worsening of the disease [48]. In this study, the antimetastasis activity of 2G_ODF was evaluated using transwell cell migration and invasion assays. 2G_ODF at IC_60_ concentration was used a representative. As shown in Figure 6a,b, the percentage of proportional invasiveness of the 2G_ODF treatment group (0.66% ± 0.58%) was significantly lower than that of the PCO40 solution (50.00% ± 2.50%) and control (100%) groups at a 95% confidence interval. This result indicates that 2G_ODF has an antimetastasis effect; this finding was consistent with that of Mandal et al., who reported that GLO exhibited antimetastasis activity by binding to estrogen receptors and through the downregulation of metastasis-associated protein-1 [10].

### 3.14. Apoptosis Analysis

**Figure 7:**
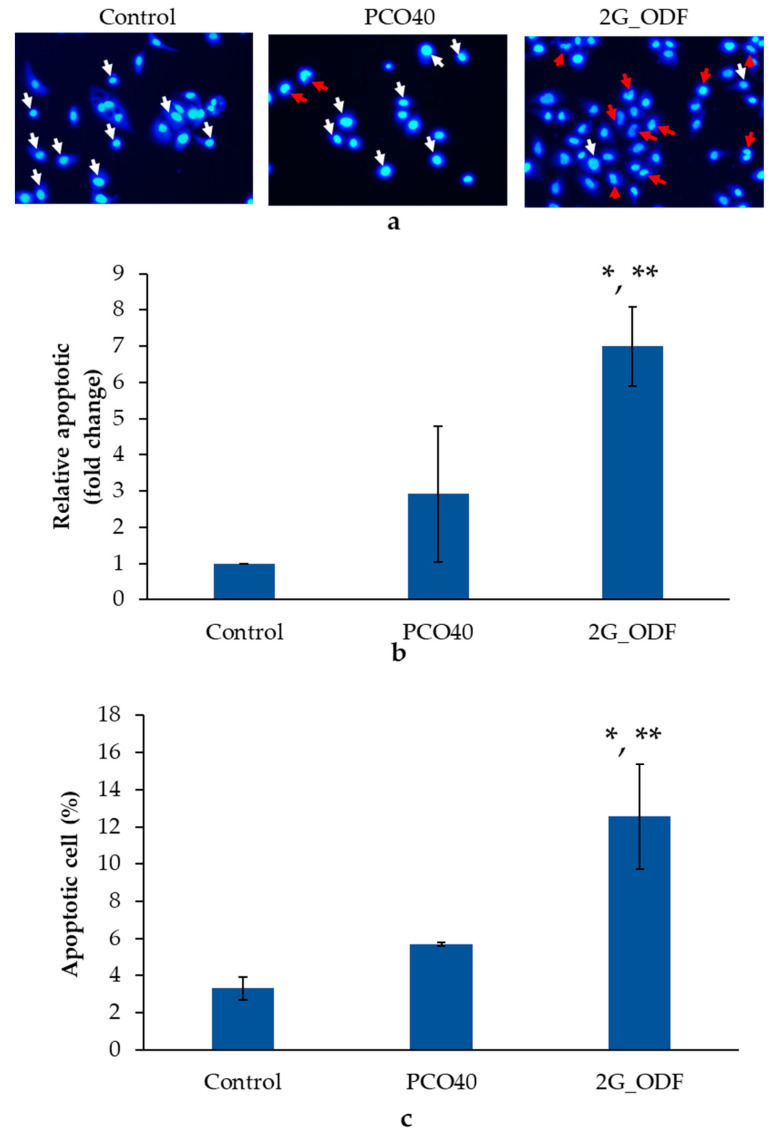
Nuclear fragmentation of KON cells after treatment with control, PCO40 solutions, and 2G_ODF at IC_60_ (**a**): Red arrow represents nuclear fragmentation and white arrow presents control nucleus. Relative apoptotic cells indicated by nuclear fragmentation assessment with Hoechst 33258 staining (**b**) and flow cytometry (**c**). The results are depicted as mean ± SD (n = 3). * denotes significant difference (*p* < 0.05) when compared with control. ** denotes significant difference (*p* < 0.05) when compared with PCO40.

Among cell death mechanisms, apoptosis is a desirable pathway for new anticancer agent candidates due to the lack of inflammation activation [49]. To assess the cell death mechanism after induction with ODFs, the nuclear fragmentation and phosphatidylserine exposure on the cell surface, which were remarkable features of apoptotic cells, were evaluated using fluorescent microscopy and flow cytometry. The results showed significant differences among the 2G_ODF, PCO40 solution, and negative control treatment groups at a 95% confidence interval. After exposure to 2G_ODF at IC_60_, an increase in the number of apoptotic cells was found, which was 6.70 ± 1.10 times higher than that in the negative control. In addition, the ODF demonstrated greater effectiveness than the PCO40 solution, as shown in Figure 7a,b.

Flow cytometry was also used to identify apoptotic cells that displayed phosphatidylserine on their cell surface, as evidenced by positive Annexin-V staining. The apoptosis of KON using flow cytometry is depicted in Figure 7c. After treatment with 2G_ODF and PCO40 solution, the percentage of apoptotic cells was 12.56% ± 2.82% and 3.31% ± 0.08%, respectively, which was significantly greater than that in the negative control group. The 2G_ODF treatment group showed a significantly higher percentage of apoptotic cells than the PCO40 solution group, supporting anticancer activity as a direct result of GLO. Nuclear fragmentation and flow cytometry results revealed that 2G_ODF can cause cell death via apoptosis. This finding was consistent with that of previous reports, which revealed that β-caryophyllene, a major bioactive in GLO, can trigger apoptosis through DNA fragmentation, caspase-3 activation [50], and an increase in the Bax/Bcl-2 ratio [51].

## 4. Conclusions

In this study, the potential use of ODFs containing GLO nanoemulsions against oral cancer cells was investigated. GLO nanoemulsions exhibited a small size of approximately 50 nm, a relatively low zeta potential, and good stability within the range of GLO:VCO ratios of 50:50 to 70:30. After incorporation into films, 2G_ODF showed a potential application prospect due to its high concentration loading of GLO with favorable mechanical properties. Moreover, our nuclear fragmentation results and positive Annexin-V staining revealed that cell death occurred via apoptosis. Furthermore, the ODFs containing GLO nanoemulsions inhibited cancer progression by effectively suppressing colony formation and cellular metastasis. In conclusion, these findings suggest that ODFs containing GLO nanoemulsions hold great promise for oral cancer treatment.

## Figures and Tables

**Table 1:** Properties of ODFs. The results are depicted as mean ± SD (n = 5; weight and n = 3; pH, thickness, moisture absorption and disintegration time).

Samples	pH	Weight (µg)	Thickness (µm)	Moisture Absorption (%)	Disintegration Time (s)
Sodium alginate film	7.11 ± 0.03	6.8 ± 1.5	59.0 ± 9.0	57.56 ± 5.00	18.11 ± 0.60
1G_ODF	6.24 ± 0.21	18.3 ± 2.8	41.3 ± 9.0	49.29 ± 6.18	12.75 ± 2.60
2G_ODF	6.06 ± 0.08	21.4 ± 3.0	44.0 ± 14.0	29.63 ± 5.71	15.25 ± 1.13
5G_ODF	5.91 ± 0.08	25.1 ± 3.0	60.7 ± 6.0	23.93 ± 7.69	17.00 ± 1.66
10G_ODF	5.84 ± 0.03	29.2 ± 5.2	77.0 ± 13.0	21.11 ± 2.51	18.83 ± 4.22
20G_ODF	5.61 ± 0.09	37.5 ± 4.4	82.5 ± 3.5	15.93 ± 5.55	21.35 ± 5.86
30G_ODF	5.46 ± 0.01	46.5 ± 7.8	121.0 ± 9.0	10.65 ± 1.20	22.75 ± 4.50

**Table 2:** β-caryophyllene content.

Sample	β-Caryophyllene Content (µg/g)
GLO	176,950.98 ± 239.55
2G_ODF	129.51 ± 3.58
5G_ODF	220.82 ± 12.04
10G_ODF	309.58 ± 15.92

**Table 3:** IC_50_ of β-caryophyllene, GLO, 2G_ODF, 5G_ODF, and 10G_ODF. The results are depicted as mean ± SD (n = 3).

Samples	IC_50_
β-caryophyllene	3.73 ± 0.58 µg/mL
GLO	11.65 ± 2.61 µg/mL
2G_ODF	62.49 ± 6.22 mg/mL
5G_ODF	24.11 ± 1.22 mg/mL
10G_ODF	15.29 ± 2.04 mg/mL

**Table 4:** Tensile strength, β-caryophyllene content, and IC_50_ of 2G_ODF before and after storage at 25 °C for 1 year. The results are depicted as mean ± SD (n = 3).

Formulation	Tensile Strength (N/mm^2^)	β-Caryophyllene Content (µg/g)	IC_50_ (mg/mL)
2G_ODF at initial	0.67 ± 0.15	129.51 ± 3.58	62.49 ± 6.22
2G_ODF after storage at 25 °C for 1 year	0.60 ± 0.24	113.05 ± 4.45	65.53 ± 5.12

## Data Availability

Data are contained within the article and supplementary materials.

## Supplementary Materials

The following are available online at https://www.mdpi.com/article/10.3390/pharmaceutics15112631/s1. Supplementary Figure S1: Standard curve of β-caryophyllene. Supplementary Figure S2: Chromatogram of β-caryophyllene. Supplementary Figure S3: Chromatogram of GLO.
