# Supplementary material for: Nanoemulsion-Based Orodispersible Film Formulation of Guava Leaf Oil for Inhibition of Oral Cancer Cells
Source: Pharmaceutics. 2023 Nov 16;15(11):2631. doi: 10.3390/pharmaceutics15112631 (PMC10675713; doi:10.3390/pharmaceutics15112631)
Supplement: Supplementary file 1 [file pharmaceutics-15-02631-s001.zip › pharmaceutics-2682047-supplementary.pdf]

pharmaceutics

Supplementary Material: Nanoemulsion-Based Orodispersible Film Formulation of Guava Leaf Oil for Inhibition of Oral Cancer Cells

Yotsanan Weerapol, Suwisit Manmuan, Tiraniti Chuenbarn, Sontaya Limmatvapirat, and Sukannika Tubtimsri

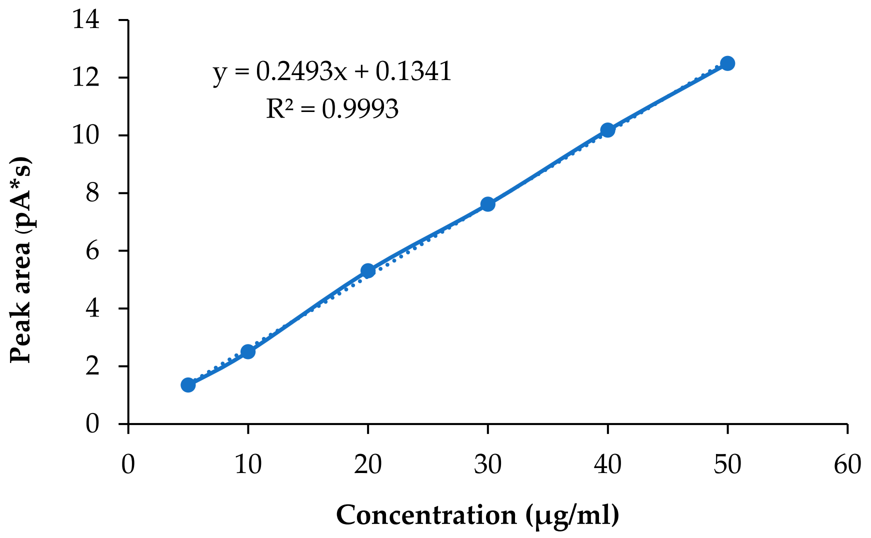

Figure S1. Standard curve of β-caryophyllene.

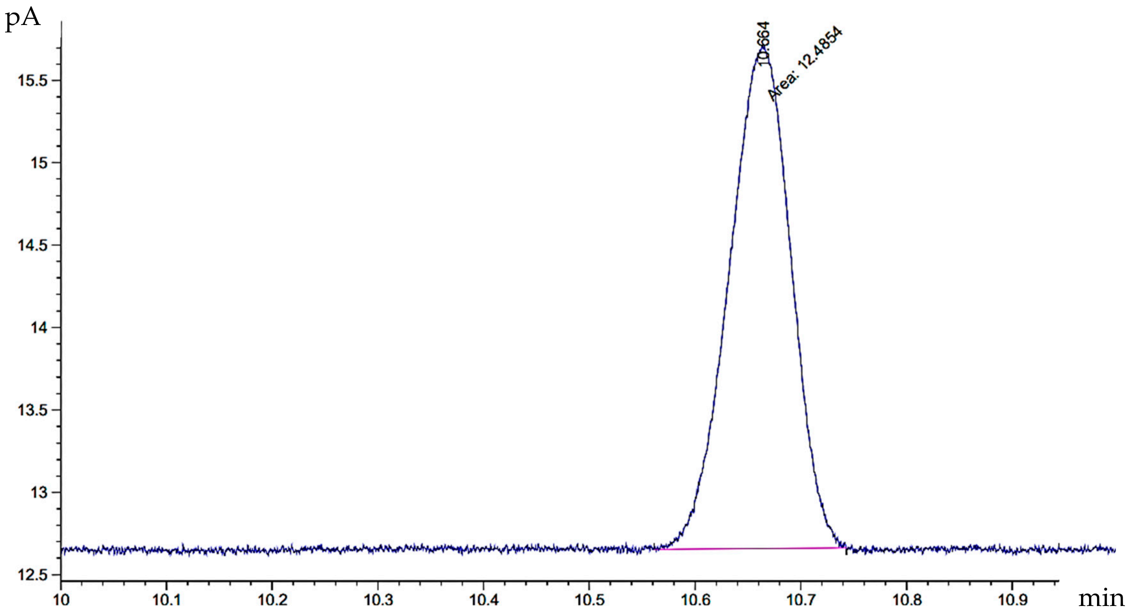

Figure S2. Chromatogram of β-caryophyllene.

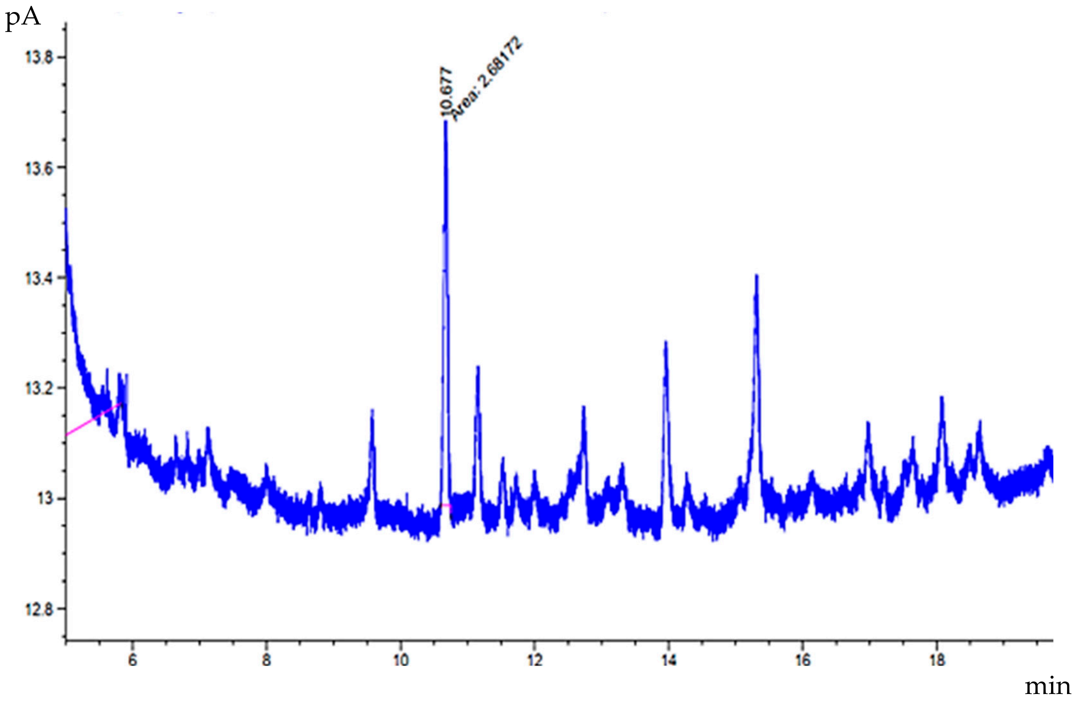

Figure S3. Chromatogram of GLO.
